# Crystal structures and Hirshfeld surface analyses of the two isotypic compounds (*E*)-1-(4-bromo­phen­yl)-2-[2,2-di­chloro-1-(4-nitro­phen­yl)ethen­yl]diazene and (*E*)-1-(4-chloro­phen­yl)-2-[2,2-di­chloro-1-(4-nitro­phen­yl)ethen­yl]diazene

**DOI:** 10.1107/S2056989019010004

**Published:** 2019-07-19

**Authors:** Mehmet Akkurt, Namiq Q. Shikhaliyev, Gulnar T. Suleymanova, Gulnare V. Babayeva, Gunay Z. Mammadova, Ayten A. Niyazova, Irada M. Shikhaliyeva, Flavien A. A. Toze

**Affiliations:** a Department of Physics, Faculty of Sciences, Erciyes University, 38039 Kayseri, Turkey; b Organic Chemistry Department, Baku State University, Z. Xalilov str. 23, Az, 1148 Baku, Azerbaijan; c Department of Chemistry, Faculty of Sciences, University of Douala, PO Box 24157, Douala, Republic of , Cameroon

**Keywords:** crystal structure, diazene derivatives, dyes, inter­molecular halogen bonds, Hirshfeld surface analysis

## Abstract

In the two isotypic title compounds, C_14_H_8_BrCl_2_N_3_O_2_, (**I**), and C_14_H_8_Cl_3_N_3_O_2_, (**II**), the substitution of one of the phenyl rings is different [Br for (**I**) and Cl for (**II**)]. Aromatic rings form dihedral angles of 60.9 (2) and 64.1 (2)°, respectively. Mol­ecules are linked through weak *X*⋯Cl contacts [*X* = Br for (**I**) and Cl for (**II**)], C—H⋯Cl and C—Cl⋯π inter­actions into sheets parallel to the *ab* plane. Additional van der Waals inter­actions consolidate the three-dimensional packing. Hirshfeld surface analysis of the crystal structures indicates that the most important contributions for the crystal packing for (**I**) are from C⋯H/H⋯C (16.1%), O⋯H/H⋯O (13.1%), Cl⋯H/H⋯Cl (12.7%), H⋯H (11.4%), Br⋯H/H⋯Br (8.9%), N⋯H/H⋯N (6.9%) and Cl⋯C/C⋯Cl (6.6%) inter­actions, and for (**II**), from Cl⋯H / H⋯Cl (21.9%), C⋯H/H⋯C (15.3%), O⋯H/H⋯O (13.4%), H⋯H (11.5%), Cl⋯C/C⋯Cl (8.3%), N⋯H/H⋯N (7.0%) and Cl⋯Cl (5.9%) inter­actions. The crystal of (I) studied was refined as an inversion twin, the ratio of components being 0.9917 (12):0.0083 (12).

## Abstract 2

In the crystals of the two isotypic compounds, mol­ecules are linked by weak halogen–halogen (Br⋯Cl or Cl⋯Cl) contacts and C—Cl⋯π inter­actions into sheets lying parallel to the *ab* plane.

## Chemical context   

Compounds with azo/hydrazone moieties are ubiquitous in various fields, ranging from organic/inorganic synthesis, catal­ysis, and medicinal chemistry to material chemistry. They are used as dyes, ligands, solvatochromic materials, mol­ecular switches, or analytical reagents amongst other applications (Akbari *et al.*, 2017 ▸; Asadov *et al.*, 2016 ▸; Gurbanov *et al.*, 2018 ▸; Kopylovich *et al.*, 2011 ▸; Ma *et al.*, 2017 ▸; Mahmoudi *et al.*, 2018 ▸; Mahmudov *et al.*, 2014 ▸, 2019 ▸).
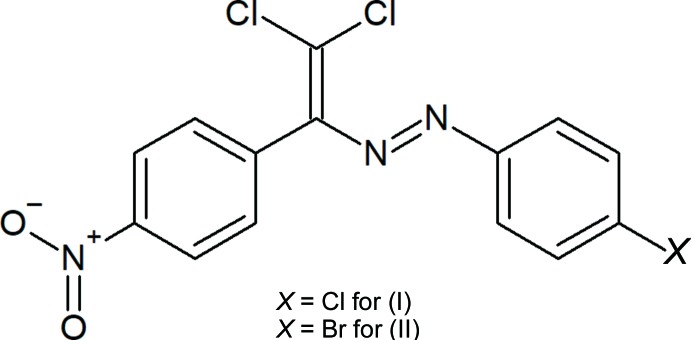


The non-covalent donor/acceptor properties of azo/hydrazones depend strongly on the attached functional groups (Shixaliyev *et al.*, 2013 ▸, 2014 ▸, 2018 ▸). In a previous study we have attached halogen atoms to dye mol­ecules, which led to halogen bonding (Maharramov *et al.*, 2018 ▸; Shixaliyev *et al.*, 2018 ▸). In a continuation of our work in this direction, we have now synthesized two new azo dyes, (*E*)-1-(4-bromo­phen­yl)-2-(2,2-di­chloro-1-(4-nitro­phen­yl)vin­yl)diazene (I) and (*E*)-1-(4-chloro­phen­yl)-2-(2,2-di­chloro-1-(4-nitro­phen­yl)vin­yl)diazene (II), and report here their mol­ecular and crystal structures.

## Structural commentary   

**Figure 1:**
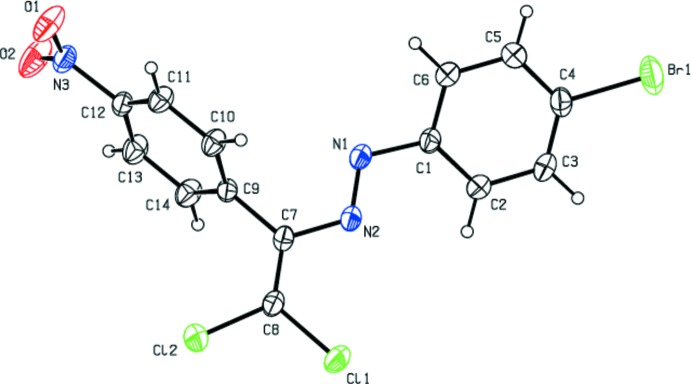
The mol­ecular structure of (I) with displacement ellipsoids drawn at the 30% probability level.

Compounds (I) and (II) are isotypic. Their mol­ecular structures (Figs. 1 ▸ and 2 ▸) are not planar. For the bromo-substituted compound (I), the dihedral angle between the essentially planar 4-bromo­phenyl ring C1–C6 [maximum deviation = 0.015 (6) Å at atom C5] and the nitro-substituted benzene ring C9–C14 [maximum deviation = −0.009 (4) Å at atom C9] is 60.9 (2)°, for the chloro-substituted compound (II) the corresponding value is 64.1 (2)°. The torsion angles involving the central diazene group amount to 18.3 (6)° for C2—C1—N1—N2, −179.1 (3)° for C1—N1—N2—C7, and 175.4 (4)° for N1—N2—C7—C8 for (I). The corresponding values for (II) are −17.0 (5)°, 179.0 (3)° and 175.4 (4)°, respectively. The bond lengths and angles are within normal ranges and are comparable to those in the related structures detailed in the *Database survey*.

## Supra­molecular features and Hirshfeld surface analysis   

**Figure 3:**
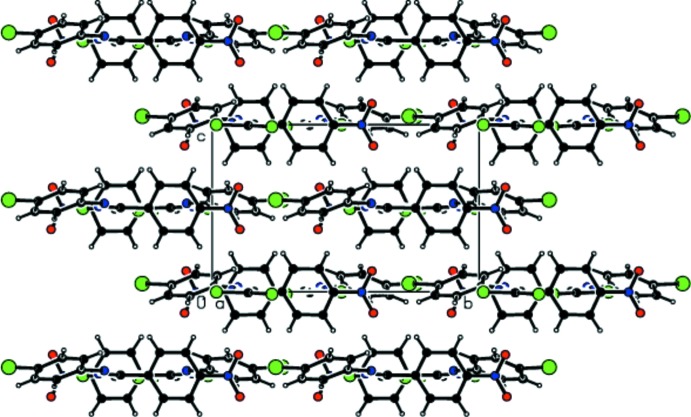
Packing in the crystal structure of (I) showing chains running parallel to the *a-*axis.

**Figure 5:**
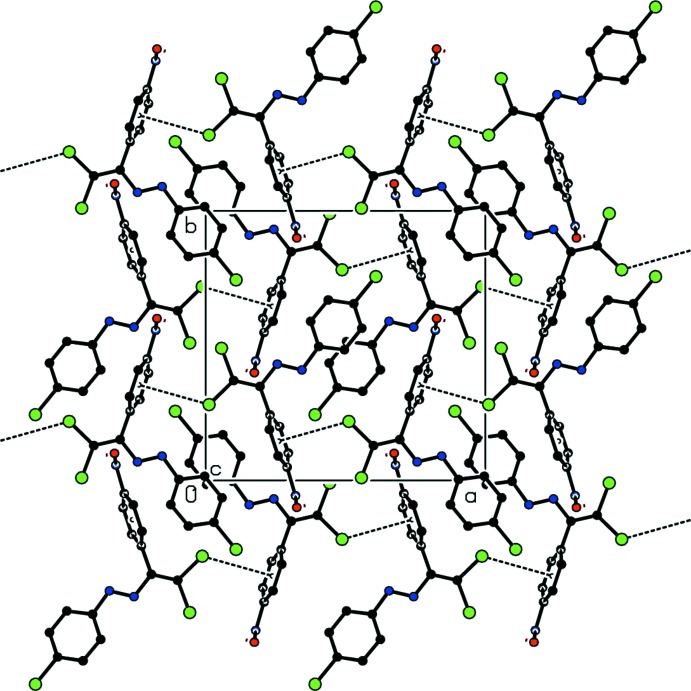
Formation of sheets in (II) parallel to *ab* through C—Cl⋯π contacts.

As a result of the isotypism of (I) and (II), the packing features are generally very similar in the two structures. Mol­ecules are linked by weak Br⋯Cl contacts [for (I)] or Cl⋯Cl contacts [for (II)] and C—H⋯Cl inter­actions into chains extending along the *a*-axis direction (Tables 1 ▸–3 ▸ ▸; Figs. 3 ▸ and 4 ▸). Additional C—Cl⋯π inter­actions lead to the formation of sheets parallel to the *ab* plane (Fig. 5 ▸). van der Waals inter­actions (Table 3 ▸) consolidate the three-dimensional packing.

Hirshfeld surface analysis (Spackman & Jayatilaka, 2009 ▸) was used to investigate the inter­molecular inter­actions in the crystal structures of both compounds (*CrystalExplorer3.1*; Wolff *et al.*, 2012 ▸). The surface plots (Spackman *et al.*, 2008 ▸) mapped over *d*_norm_ were generated to qu­antify and visualize the inter­molecular inter­actions and to explain the observed crystal packing. Dark-red spots on the *d*_norm_ surface arise as a result of short inter­atomic contacts (Tables 1 ▸–3 ▸ ▸), while the other weaker inter­molecular inter­actions appear as light-red spots.

**Figure 6:**
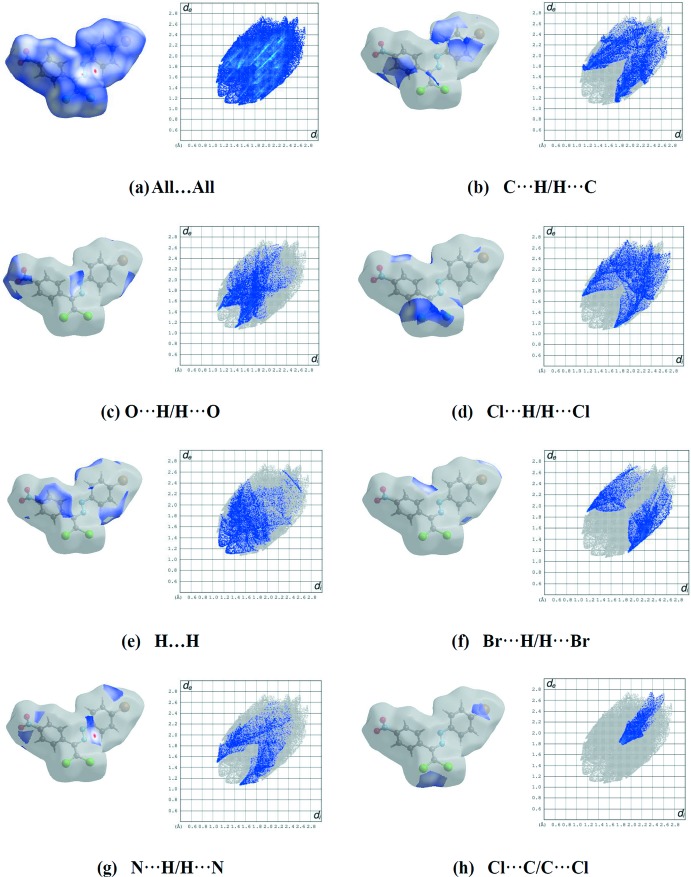
Hirshfeld surface representations and full two-dimensional fingerprint plots for (I), showing (*a*) all inter­actions, and delineated into (*b*) C⋯H/H⋯C (*c*), O⋯H/H⋯O (*d*), Cl⋯H/H⋯Cl (*e*), H⋯H (*f*), Br⋯H/H⋯Br (*g*), N⋯H/H⋯N and (*h*) Cl⋯C/C⋯Cl inter­actions. The *d*_i_ and *d*_e_ values are the closest inter­nal and external distances (in Å) from a given point on the Hirshfeld surface.

For (I), the red points, which represent closer contacts and negative *d*_norm_ values on the surface, correspond to the C—H⋯O inter­actions. The reciprocal O⋯H/H⋯O inter­actions appear as two symmetrical broad wings in the two-dimensional fingerprint plots with *d*_e_ + *d*_i_ ≃ 2.5 Å and contribute 13.1% to the Hirshfeld surface (Fig. 6 ▸ *b*). The reciprocal Cl⋯H/H⋯Cl inter­action with a contribution of 13.8% is present as sharp symmetrical spikes at *d*_e_ + *d*_i_ ≃ 2.8 Å (Fig. 6 ▸ *c*).

**Figure 7:**
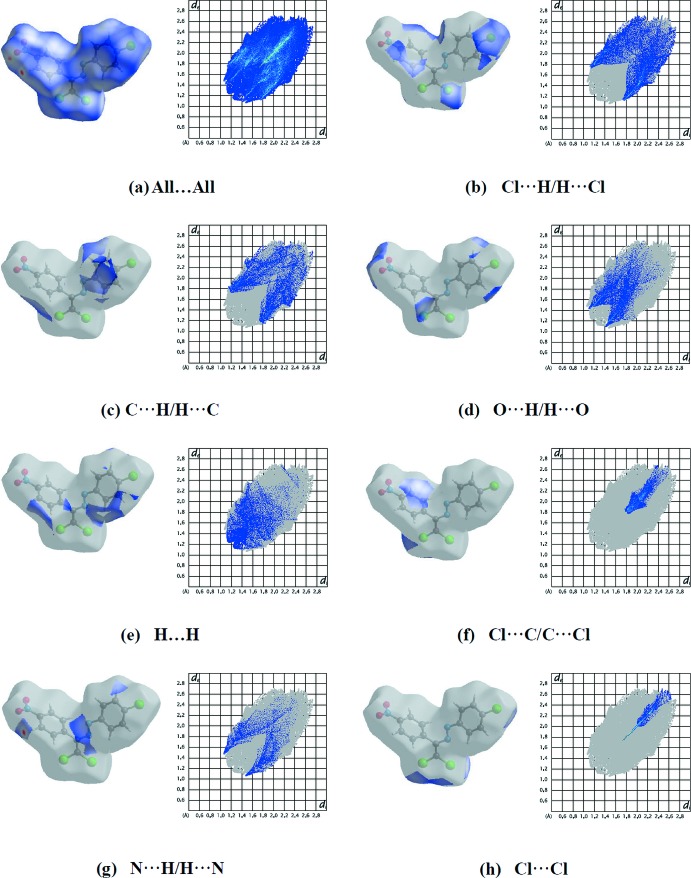
The Hirshfeld surface representations and the full two-dimensional fingerprint plots for (II), showing (*a*) all inter­actions, and delineated into (*b*) Cl⋯H/H⋯Cl, (*c*) C⋯H/H⋯C, (*d*) O⋯H/H⋯O, (*e*) H⋯H, (*f*) Cl⋯C/C⋯Cl, (*g*) N⋯H/H⋯N and (*h*) Cl⋯Cl inter­actions. The *d*_i_ and *d*_e_ values are the closest inter­nal and external distances (in Å) from a given point on the Hirshfeld surface.

For (II), the percentage contributions of various contacts to the total Hirshfeld surface are shown in the two-dimensional fingerprint plots in Fig. 7 ▸. The reciprocal Cl⋯H/H⋯Cl inter­actions appear as two symmetrical broad wings with *d*_e_ + *d*_i_ ≃ 2.9 Å and contribute 21.9% to the Hirshfeld surface (Fig. 7 ▸ *b*). The reciprocal C⋯H/H⋯C and O⋯H/H⋯O inter­actions (15.3, 13.4% contributions, respectively) are present as sharp symmetrical spikes at *d*_e_ + *d*_i_ ≃ 2.95 and 2.5 Å, respectively (Fig. 7 ▸ *c*–*d*). The small percentage contributions of both compounds to the Hirshfeld surfaces from the various other inter­atomic contacts are comparatively listed in Table 4 ▸. Although there is almost agreement on the values given for the mol­ecules of (I) and (II), some differences are due to the different halogen atoms substituting the phenyl ring and the different mol­ecular environment in the crystal structures.

## Database survey   

A search of the Cambridge Structural Database (CSD, Version 5.40, November 2018; Groom *et al.*, 2016 ▸) for structures having an (*E*)-1-(2,2-di­chloro-1-phenyl­vin­yl)-2-phenyl­diazene unit gave 23 hits. Four compounds closely resemble the title compound, *viz*. 1-(4-chloro­phen­yl)-2-[2,2-di­chloro-1-(4-fluoro­phen­yl)ethen­yl]diazene (CSD refcode HODQAV; Shikhaliyev *et al.*, 2019 ▸), 1-[2,2-di­chloro-1-(4-nitro­phen­yl)ethen­yl]-2-(4-fluoro­phen­yl)diazene (XIZREG; Atioğlu *et al.*, 2019 ▸), 1,1-[methyl­enebis(4,1-phenyl­ene)]bis­[(2, 2-di­chloro-1-(4-nitro­phen­yl)ethen­yl]diazene (LEQXIR; Shixaliyev *et al.*, 2018 ▸), 1,1-[methyl­enebis(4,1-phenyl­ene)]bis­{[2,2-di­chloro-1-(4-chloro­phen­yl)ethen­yl]diazene} (LEQXOX; Shixaliyev *et al.*, 2018 ▸),

In the crystal of HODQAV, mol­ecules are stacked in columns along the *a* axis *via* weak C—H⋯Cl hydrogen bonds and face-to-face π–π stacking inter­actions. The crystal packing is further stabilized by short Cl⋯Cl contacts. In XIZREG, mol­ecules are linked by C—H⋯O hydrogen bonds into zigzag chains running along the *c*-axis direction. The crystal packing is further stabilized by C—Cl⋯π, C—F⋯π and N—O⋯π inter­actions. In the crystal of LEQXIR, C—H⋯N and C—H⋯O hydrogen bonds and Cl⋯O contacts were found, and in LEQXOX, C—H⋯N and Cl⋯Cl contacts are observed.

## Synthesis and crystallization   

Dyes (I) and (II) were synthesized according to a literature protocol (Shixaliyev *et al.*, 2018 ▸). For (I), a 20 ml screw neck vial was charged with DMSO (10 ml), (*E*)-1-(4-bromo­phen­yl)-2-(4-nitro­benzyl­idene)hydrazine (320 mg, 1 mmol), tetra­methyl­ethylenedi­amine (TMEDA) (295 mg, 2.5 mmol), CuCl (2 mg, 0.02 mmol) and CCl_4_ (20 mmol, 10 equiv.). After 1–3 h (until TLC analysis showed complete consumption of the corresponding Schiff base), the reaction mixture was poured into 0.01 *M* solution of HCl (100 ml, pH ∼2-3), and extracted with di­chloro­methane (3 × 20 ml). The organic phases were combined and washed with water (3 × 50 ml), brine (30 ml), dried over anhydrous Na_2_SO_4_ and concentrated *in vacuo* in a rotary evaporator. The residue was purified by column chromatography on silica gel using appropriate mixtures of hexane and di­chloro­methane (*v*/*v*: 3/1–1/1). An orange solid was obtained (yield 58%); mp 418 K. Analysis calculated for C_14_H_8_BrCl_2_N_3_O_2_ (*M =* 401.04): C, 41.93; H, 2.01; N, 10.48; found: C, 41.87; H, 2.03; N, 10.39%. ^1^H NMR (300 MHz, CDCl_3_) *δ* 8.30 (*d*, 2H, *J* = 9.02 Hz), 7.65–7.56 (*m*, 4H), 7.38 (*d*, 2H, *J* = 9.24Hz). ^13^C NMR (75 MHz, CDCl_3_) *δ* 151.26, 150.60, 147.97, 139.21, 137.18, 132.49, 131.28, 126.83, 124.72, 123.44. ESI–MS: *m*/*z*: 402.08 [*M* + H]^+^.

For (II), the procedure was the same as that for (I) using (*E*)-1-(4-chloro­ophen­yl)-2-(4-nitro­benzyl­idene)hydrazine (276 mg, 1 mmol). An orange solid was obtained (yield 64%); mp 448 K. Analysis calculated for C_14_H_8_Cl_3_N_3_O_2_ (*M* = 356.59): C, 47.16; H, 2.26; N, 11.78; found: C, 47.09; H, 2.23; N, 11.65%. ^1^H NMR (300 MHz, CDCl3) δ 8.32–7.37 (8H, Ar). ^13^C NMR (75 MHz, CDCl_3_) δ 150.91, 150.55, 147.98, 139.28, 138.22, 137.02, 131.24, 129.49, 124.52, 123.44. ESI–MS: *m*/*z*: 357.70 [*M* + H]+.

Compounds (I) and (II) were dissolved in di­chloro­methane and then left at room temperature for slow evaporation; orange crystals of both compounds suitable for X-rays started to form after *ca* 2 d.

## Refinement   

Crystal data collection and structure refinement details are summarized in Table 5 ▸. C-bound H atoms were constrained to ideal values with C—H = 0.93 Å and with *U*_iso_(H) = 1.2*U*_eq_(C). The crystal of (I) studied was refined as an inversion twin, the ratio of components being 0.9917 (12):0.0083 (12).

## Supplementary Material

Crystal structure: contains datablock(s) I, II, global. DOI: 10.1107/S2056989019010004/wm5507sup1.cif

Structure factors: contains datablock(s) I. DOI: 10.1107/S2056989019010004/wm5507Isup2.hkl

Structure factors: contains datablock(s) II. DOI: 10.1107/S2056989019010004/wm5507IIsup3.hkl

Supporting information file. DOI: 10.1107/S2056989019010004/wm5507Isup4.cml

Supporting information file. DOI: 10.1107/S2056989019010004/wm5507IIsup5.cml

CCDC references: 1940144, 1940145

Additional supporting information:  crystallographic information; 3D view; checkCIF report

## Figures and Tables

**Figure 2:**
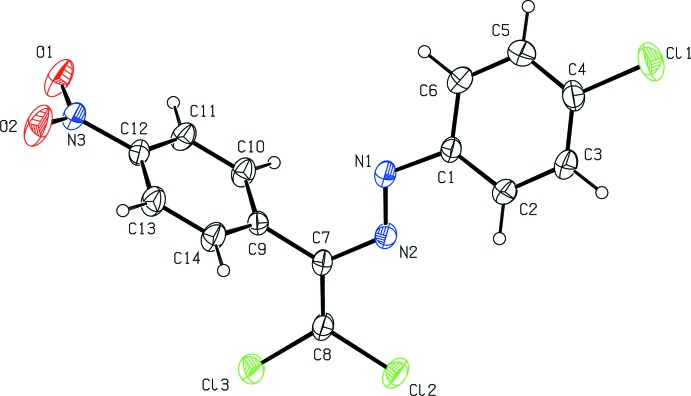
The mol­ecular structure of (II) with displacement ellipsoids drawn at the 30% probability level.

**Figure 4:**
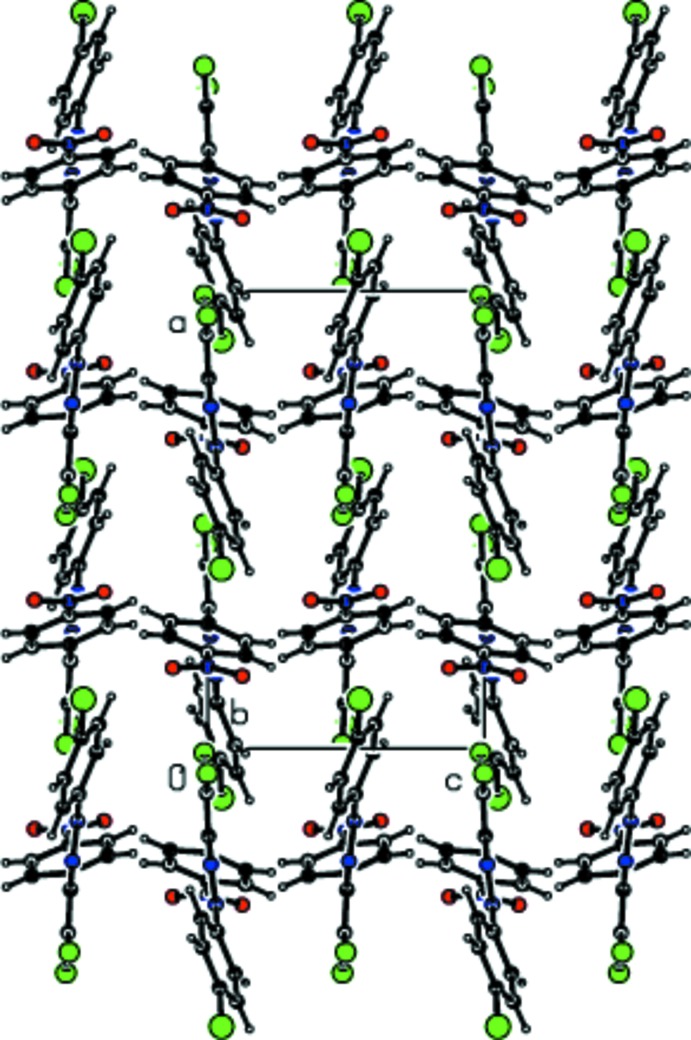
A view of the packing in (I) along the *a* axis showing C—H⋯Cl contacts.

**Table 1:** Hydrogen-bond geometry (Å, °) for (I) *Cg*2 is the centroid of the C9–C14 ring.

*D*—H⋯*A*	*D*—H	H⋯*A*	*D*⋯*A*	*D*—H⋯*A*
C6—H6⋯Cl2^i^	0.93	2.92	3.593 (5)	131
C8—Cl2⋯*Cg*2^ii^	1.71 (1)	3.66 (1)	4.710 (5)	118 (1)

Symmetry codes: (i) 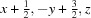; (ii) 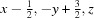.

**Table 2:** C—Cl⋯π geometry (Å, °) for (II) *Cg*2 is the centroid of the C9–C14 ring.

C—C⋯π	C—Cl	Cl⋯π	C⋯π	C—C⋯π
C8—Cl3⋯*Cg*2^i^	1.71 (1)	3.62 (1)	4.703 (3)	120 (1)

Symmetry code: (i) 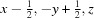.

**Table 3:** Summary of short inter­atomic contacts (Å) in the crystal structures of compounds (I) and (II)

Contact	Distance	Symmetry operation
Compound (I)		
H10⋯Br1	3.18	1 − *x*, 1 − *y*, 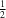 + *z*
Br1⋯Cl1	3.5125 (12)	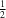 + *x*, 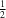 − *y*, *z*
H2⋯H11	2.54	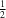 − *x*, − 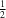 + *y*, − 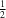 + *z*
Cl2⋯H6	2.92	− 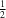 + *x*, 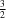 − *y*, *z*
O2⋯H3	2.68	*x*, 1 + *y*, *z*
H13⋯N2	2.73	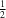 − *x*, 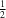 + *y*, − 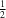 + *z*
Compound (II)		
H10⋯Cl1	3.13	2 − *x*, −*y*, − 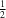 + *z*
Cl1⋯Cl2	3.4847 (14)	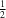 + *x*, − 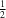 − *y*, *z*
H2⋯H11	2.56	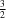 − *x*, − 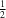 + *y*, 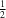 + *z*
Cl3⋯H6	2.98	− 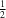 + *x*, 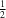 − *y*, *z*
O2⋯H3	2.66	*x*, 1 + *y*, *z*
H13⋯N2	2.69	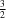 − *x*, 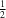 + *y*, 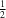 + *z*

**Table 4:** Percentage contributions of inter­atomic contacts to the Hirshfeld surface in the crystal structures of compounds (I) and (II)

Contact	(I)	(II)
C⋯H/H⋯C	16.1	15.3
O⋯H/H⋯O	13.1	13.4
Cl⋯H/H⋯Cl	12.7	21.9
H⋯H	11.4	11.5
Br⋯H/H⋯Br	8.9	–
N⋯H/H⋯N	6.9	7.0
Cl⋯C/C⋯Cl	6.6	8.3
Cl⋯Br/Br⋯Cl	5.2	–
Cl⋯O/O⋯Cl	4.9	5.8
O⋯C/C⋯O	3.8	3.9
Cl⋯N/N⋯Cl	3.4	3.4
C⋯C	2.1	2.3
Br⋯C/C⋯Br	1.5	–
Br⋯O/O⋯Br	1.2	–
N⋯O/O⋯N	1.1	1.0
Cl⋯Cl	1.0	5.9
N⋯C/C⋯N	0.1	0.2
Br⋯N/N⋯Br	0.1	–

**Table 5:** Experimental details

	(I)	(II)
Crystal data		
Chemical formula	C_14_H_8_BrCl_2_N_3_O_2_	C_14_H_8_Cl_3_N_3_O_2_
*M*_r_	401.04	356.58
Crystal system, space group	Orthorhombic, *P* *n* *a*2_1_	Orthorhombic, *P* *n* *a*2_1_
Temperature (K)	296	296
*a*, *b*, *c* (Å)	13.9181 (7), 13.4336 (6), 8.4080 (4)	13.8689 (7), 13.3674 (7), 8.3620 (5)
*V* (Å^3^)	1572.05 (13)	1550.24 (15)
*Z*	4	4
Radiation type	Mo *K*α	Mo *K*α
μ (mm^−1^)	2.96	0.60
Crystal size (mm)	0.19 × 0.14 × 0.08	0.17 × 0.14 × 0.07

Data collection		
Diffractometer	Bruker APEXII CCD	Bruker APEXII CCD
Absorption correction	Multi-scan (*SADABS*; Bruker, 2003 ▸)	Multi-scan (*SADABS*; Bruker, 2003 ▸)
*T*_min_, *T*_max_	0.608, 0.784	0.911, 0.946
No. of measured, independent and observed [*I* > 2σ(*I*)] reflections	23012, 3429, 2811	11687, 3156, 2547
*R*_int_	0.057	0.038
(sin θ/λ)_max_ (Å^−1^)	0.641	0.626

Refinement		
*R*[*F* ^2^ > 2σ(*F* ^2^)], *wR*(*F* ^2^), *S*	0.033, 0.081, 1.02	0.037, 0.091, 1.04
No. of reflections	3429	3156
No. of parameters	200	199
No. of restraints	1	1
H-atom treatment	H-atom parameters constrained	H-atom parameters constrained
Δρ_max_, Δρ_min_ (e Å^−3^)	0.31, −0.50	0.18, −0.25
Absolute structure	Refined as an inversion twin	Flack *x* determined using 1011 quotients [(*I* ^+^)−(*I* ^−^)]/[(*I* ^+^)+(*I* ^−^)] (Parsons *et al.*, 2013 ▸).
Absolute structure parameter	0.008 (13)	0.14 (3)

Computer programs: *APEX3* and *SAINT* (Bruker, 2007 ▸), *SHELXT2016/6* (Sheldrick, 2015*a* ▸), *SHELXL2016/6* (Sheldrick, 2015*b* ▸), *ORTEP-3 for Windows* (Farrugia, 2012 ▸) and *PLATON* (Spek, 2009 ▸).

## supplementary crystallographic information

### (E)-1-(4-Bromophenyl)-2-[2,2-dichloro-1-(4-nitrophenyl)ethenyl]diazene (I) Crystal data

**Table:**

C_14_H_8_BrCl_2_N_3_O_2_	*D*_x_ = 1.694 Mg m^−^^3^
*M**_r_* = 401.04	Mo *K*α radiation, λ = 0.71073 Å
Orthorhombic, *P**n**a*2_1_	Cell parameters from 8656 reflections
*a* = 13.9181 (7) Å	θ = 2.9–27.0°
*b* = 13.4336 (6) Å	µ = 2.96 mm^−^^1^
*c* = 8.4080 (4) Å	*T* = 296 K
*V* = 1572.05 (13) Å^3^	Plate, orange
*Z* = 4	0.19 × 0.14 × 0.08 mm
*F*(000) = 792	

### (E)-1-(4-Bromophenyl)-2-[2,2-dichloro-1-(4-nitrophenyl)ethenyl]diazene (I) Data collection

**Table:**

Bruker APEXII CCD diffractometer	2811 reflections with *I* > 2σ(*I*)
φ and ω scans	*R*_int_ = 0.057
Absorption correction: multi-scan (SADABS; Bruker, 2003)	θ_max_ = 27.1°, θ_min_ = 2.9°
*T*_min_ = 0.608, *T*_max_ = 0.784	*h* = −17→17
23012 measured reflections	*k* = −14→17
3429 independent reflections	*l* = −10→10

### (E)-1-(4-Bromophenyl)-2-[2,2-dichloro-1-(4-nitrophenyl)ethenyl]diazene (I) Refinement

**Table:**

Refinement on *F*^2^	Hydrogen site location: inferred from neighbouring sites
Least-squares matrix: full	H-atom parameters constrained
*R*[*F*^2^ > 2σ(*F*^2^)] = 0.033	*w* = 1/[σ^2^(*F*_o_^2^) + (0.0253*P*)^2^ + 0.7719*P*] where *P* = (*F*_o_^2^ + 2*F*_c_^2^)/3
*wR*(*F*^2^) = 0.081	(Δ/σ)_max_ < 0.001
*S* = 1.02	Δρ_max_ = 0.31 e Å^−^^3^
3429 reflections	Δρ_min_ = −0.50 e Å^−^^3^
200 parameters	Absolute structure: Refined as an inversion twin
1 restraint	Absolute structure parameter: 0.008 (13)

### (E)-1-(4-Bromophenyl)-2-[2,2-dichloro-1-(4-nitrophenyl)ethenyl]diazene (I) Special details

**Table:**

Geometry. All esds (except the esd in the dihedral angle between two l.s. planes) are estimated using the full covariance matrix. The cell esds are taken into account individually in the estimation of esds in distances, angles and torsion angles; correlations between esds in cell parameters are only used when they are defined by crystal symmetry. An approximate (isotropic) treatment of cell esds is used for estimating esds involving l.s. planes.
Refinement. Refined as a 2-component inversion twin.

### (E)-1-(4-Bromophenyl)-2-[2,2-dichloro-1-(4-nitrophenyl)ethenyl]diazene (I) Fractional atomic coordinates and isotropic or equivalent isotropic displacement parameters (Å^2^)

**Table:**

	*x*	*y*	*z*	*U*_iso_*/*U*_eq_	
C1	0.4004 (2)	0.5082 (3)	0.5333 (5)	0.0393 (8)	
C2	0.3711 (3)	0.4164 (3)	0.4764 (6)	0.0478 (10)	
H2	0.309463	0.408665	0.435584	0.057*	
C3	0.4334 (3)	0.3360 (3)	0.4803 (6)	0.0545 (11)	
H3	0.414420	0.274430	0.440786	0.065*	
C4	0.5228 (3)	0.3483 (3)	0.5428 (6)	0.0481 (9)	
C5	0.5526 (3)	0.4381 (4)	0.6048 (7)	0.0582 (13)	
H5	0.613115	0.444124	0.650896	0.070*	
C6	0.4915 (3)	0.5191 (3)	0.5976 (6)	0.0539 (12)	
H6	0.511337	0.580727	0.635893	0.065*	
C7	0.1912 (2)	0.6569 (2)	0.5030 (5)	0.0378 (8)	
C8	0.0975 (3)	0.6351 (3)	0.4948 (6)	0.0477 (10)	
C9	0.2272 (3)	0.7616 (2)	0.5010 (5)	0.0345 (7)	
C10	0.2658 (3)	0.8034 (3)	0.6372 (5)	0.0437 (9)	
H10	0.270870	0.765504	0.729433	0.052*	
C11	0.2966 (3)	0.9009 (3)	0.6369 (5)	0.0442 (9)	
H11	0.321785	0.929565	0.728509	0.053*	
C12	0.2896 (2)	0.9547 (3)	0.4988 (5)	0.0374 (8)	
C13	0.2538 (4)	0.9149 (3)	0.3622 (5)	0.0511 (11)	
H13	0.250600	0.952658	0.269651	0.061*	
C14	0.2221 (3)	0.8170 (3)	0.3637 (5)	0.0491 (10)	
H14	0.197279	0.788772	0.271406	0.059*	
N1	0.3411 (2)	0.5954 (2)	0.5291 (5)	0.0427 (7)	
N2	0.2537 (2)	0.5747 (2)	0.5107 (4)	0.0403 (7)	
N3	0.3201 (2)	1.0595 (2)	0.4987 (5)	0.0483 (8)	
O1	0.3418 (3)	1.0978 (3)	0.6245 (5)	0.0769 (11)	
O2	0.3240 (4)	1.1036 (3)	0.3734 (5)	0.0863 (14)	
Cl1	0.05489 (8)	0.51572 (8)	0.4988 (2)	0.0743 (4)	
Cl2	0.00955 (8)	0.72322 (9)	0.4870 (2)	0.0713 (4)	
Br1	0.60741 (4)	0.23804 (4)	0.54965 (11)	0.0809 (2)	

### (E)-1-(4-Bromophenyl)-2-[2,2-dichloro-1-(4-nitrophenyl)ethenyl]diazene (I) Atomic displacement parameters (Å^2^)

**Table:**

	*U*^11^	*U*^22^	*U*^33^	*U*^12^	*U*^13^	*U*^23^
C1	0.0435 (18)	0.0297 (16)	0.045 (2)	0.0001 (13)	0.0007 (18)	0.0047 (18)
C2	0.045 (2)	0.038 (2)	0.060 (3)	−0.0022 (16)	−0.0050 (19)	−0.007 (2)
C3	0.060 (3)	0.0298 (19)	0.074 (3)	−0.0012 (17)	−0.002 (2)	−0.006 (2)
C4	0.051 (2)	0.0365 (18)	0.057 (3)	0.0094 (15)	0.006 (2)	0.008 (2)
C5	0.043 (2)	0.046 (3)	0.085 (4)	0.0025 (18)	−0.010 (2)	0.002 (2)
C6	0.048 (2)	0.036 (2)	0.077 (3)	−0.0035 (16)	−0.011 (2)	−0.003 (2)
C7	0.0440 (18)	0.0276 (15)	0.042 (2)	0.0021 (14)	0.0025 (17)	0.0016 (16)
C8	0.046 (2)	0.0290 (18)	0.068 (3)	−0.0009 (14)	0.005 (2)	−0.0031 (18)
C9	0.0358 (17)	0.0277 (16)	0.0399 (19)	0.0020 (12)	0.0015 (15)	0.0014 (16)
C10	0.058 (3)	0.035 (2)	0.038 (2)	−0.0045 (18)	−0.0006 (18)	0.0050 (17)
C11	0.056 (2)	0.038 (2)	0.039 (2)	−0.0060 (18)	−0.0025 (18)	−0.0039 (17)
C12	0.0395 (17)	0.0273 (16)	0.045 (2)	0.0006 (13)	0.0045 (17)	0.0003 (17)
C13	0.069 (3)	0.039 (2)	0.046 (2)	−0.004 (2)	−0.006 (2)	0.0098 (18)
C14	0.065 (3)	0.041 (2)	0.041 (2)	−0.007 (2)	−0.009 (2)	0.0037 (18)
N1	0.0446 (16)	0.0288 (14)	0.055 (2)	−0.0015 (11)	0.0023 (16)	0.0018 (15)
N2	0.0437 (15)	0.0280 (13)	0.0493 (19)	0.0012 (12)	0.0009 (15)	0.0017 (14)
N3	0.0564 (19)	0.0302 (16)	0.058 (2)	−0.0046 (14)	0.0018 (19)	0.0012 (18)
O1	0.119 (3)	0.041 (2)	0.071 (2)	−0.022 (2)	−0.004 (2)	−0.0088 (18)
O2	0.142 (4)	0.044 (2)	0.074 (3)	−0.028 (2)	−0.011 (2)	0.0190 (19)
Cl1	0.0508 (6)	0.0360 (5)	0.1362 (12)	−0.0104 (4)	0.0112 (8)	−0.0047 (7)
Cl2	0.0438 (5)	0.0440 (6)	0.1260 (13)	0.0068 (4)	0.0004 (7)	−0.0025 (7)
Br1	0.0781 (3)	0.0554 (3)	0.1093 (5)	0.0306 (2)	0.0097 (4)	0.0087 (4)

### (E)-1-(4-Bromophenyl)-2-[2,2-dichloro-1-(4-nitrophenyl)ethenyl]diazene (I) Geometric parameters (Å, º)

**Table:**

C1—C2	1.384 (5)	C8—Cl1	1.710 (4)
C1—C6	1.385 (6)	C9—C14	1.376 (6)
C1—N1	1.434 (4)	C9—C10	1.384 (6)
C2—C3	1.386 (6)	C10—C11	1.378 (6)
C2—H2	0.9300	C10—H10	0.9300
C3—C4	1.361 (6)	C11—C12	1.371 (6)
C3—H3	0.9300	C11—H11	0.9300
C4—C5	1.377 (6)	C12—C13	1.361 (6)
C4—Br1	1.893 (4)	C12—N3	1.470 (4)
C5—C6	1.384 (6)	C13—C14	1.387 (6)
C5—H5	0.9300	C13—H13	0.9300
C6—H6	0.9300	C14—H14	0.9300
C7—C8	1.338 (5)	N1—N2	1.257 (4)
C7—N2	1.407 (4)	N3—O2	1.209 (5)
C7—C9	1.493 (4)	N3—O1	1.214 (5)
C8—Cl2	1.705 (4)		
			
C2—C1—C6	120.0 (4)	C14—C9—C10	119.7 (3)
C2—C1—N1	123.3 (3)	C14—C9—C7	120.1 (3)
C6—C1—N1	116.7 (3)	C10—C9—C7	120.2 (3)
C1—C2—C3	120.1 (4)	C11—C10—C9	120.3 (4)
C1—C2—H2	120.0	C11—C10—H10	119.8
C3—C2—H2	120.0	C9—C10—H10	119.8
C4—C3—C2	119.1 (4)	C12—C11—C10	118.7 (4)
C4—C3—H3	120.4	C12—C11—H11	120.6
C2—C3—H3	120.4	C10—C11—H11	120.6
C3—C4—C5	121.9 (4)	C13—C12—C11	122.2 (3)
C3—C4—Br1	119.0 (3)	C13—C12—N3	118.8 (4)
C5—C4—Br1	119.1 (3)	C11—C12—N3	119.0 (4)
C4—C5—C6	119.1 (4)	C12—C13—C14	118.8 (4)
C4—C5—H5	120.4	C12—C13—H13	120.6
C6—C5—H5	120.4	C14—C13—H13	120.6
C5—C6—C1	119.8 (4)	C9—C14—C13	120.3 (4)
C5—C6—H6	120.1	C9—C14—H14	119.9
C1—C6—H6	120.1	C13—C14—H14	119.9
C8—C7—N2	115.7 (3)	N2—N1—C1	112.3 (3)
C8—C7—C9	122.2 (3)	N1—N2—C7	115.5 (3)
N2—C7—C9	122.2 (3)	O2—N3—O1	122.7 (3)
C7—C8—Cl2	123.3 (3)	O2—N3—C12	118.8 (4)
C7—C8—Cl1	122.8 (3)	O1—N3—C12	118.5 (4)
Cl2—C8—Cl1	113.8 (2)		
			
C6—C1—C2—C3	−1.5 (7)	C7—C9—C10—C11	−178.3 (4)
N1—C1—C2—C3	178.2 (4)	C9—C10—C11—C12	−0.9 (7)
C1—C2—C3—C4	1.0 (7)	C10—C11—C12—C13	−0.4 (7)
C2—C3—C4—C5	1.1 (8)	C10—C11—C12—N3	178.4 (4)
C2—C3—C4—Br1	179.5 (4)	C11—C12—C13—C14	1.0 (7)
C3—C4—C5—C6	−2.7 (8)	N3—C12—C13—C14	−177.9 (4)
Br1—C4—C5—C6	179.0 (4)	C10—C9—C14—C13	−1.2 (7)
C4—C5—C6—C1	2.1 (8)	C7—C9—C14—C13	178.8 (4)
C2—C1—C6—C5	0.0 (7)	C12—C13—C14—C9	−0.1 (7)
N1—C1—C6—C5	−179.8 (4)	C2—C1—N1—N2	18.3 (6)
N2—C7—C8—Cl2	179.2 (3)	C6—C1—N1—N2	−161.9 (4)
C9—C7—C8—Cl2	−1.9 (7)	C1—N1—N2—C7	−179.1 (3)
N2—C7—C8—Cl1	1.8 (6)	C8—C7—N2—N1	−175.4 (4)
C9—C7—C8—Cl1	−179.4 (3)	C9—C7—N2—N1	5.7 (5)
C8—C7—C9—C14	−70.7 (6)	C13—C12—N3—O2	−9.3 (6)
N2—C7—C9—C14	108.1 (5)	C11—C12—N3—O2	171.8 (5)
C8—C7—C9—C10	109.3 (5)	C13—C12—N3—O1	171.9 (5)
N2—C7—C9—C10	−71.9 (5)	C11—C12—N3—O1	−7.0 (6)
C14—C9—C10—C11	1.7 (7)		

### (E)-1-(4-Bromophenyl)-2-[2,2-dichloro-1-(4-nitrophenyl)ethenyl]diazene (I) Hydrogen-bond geometry (Å, º)

*Cg*2 is the centroid of the C9–C14 ring.

**Table:**

*D*—H···*A*	*D*—H	H···*A*	*D*···*A*	*D*—H···*A*
C6—H6···Cl2^i^	0.93	2.92	3.593 (5)	131
C8—Cl2···*Cg*2^ii^	1.71 (1)	3.66 (1)	4.710 (5)	118 (1)

Symmetry codes: (i) *x*+1/2, −*y*+3/2, *z*; (ii) *x*−1/2, −*y*+3/2, *z*.

### (E)-1-(4-Chlorophenyl)-2-[2,2-dichloro-1-(4-nitrophenyl)ethenyl]diazene (II) Crystal data

**Table:**

C_14_H_8_Cl_3_N_3_O_2_	*D*_x_ = 1.528 Mg m^−^^3^
*M**_r_* = 356.58	Mo *K*α radiation, λ = 0.71073 Å
Orthorhombic, *P**n**a*2_1_	Cell parameters from 4206 reflections
*a* = 13.8689 (7) Å	θ = 2.9–26.4°
*b* = 13.3674 (7) Å	µ = 0.60 mm^−^^1^
*c* = 8.3620 (5) Å	*T* = 296 K
*V* = 1550.24 (15) Å^3^	Prisme, orange
*Z* = 4	0.17 × 0.14 × 0.07 mm
*F*(000) = 720	

### (E)-1-(4-Chlorophenyl)-2-[2,2-dichloro-1-(4-nitrophenyl)ethenyl]diazene (II) Data collection

**Table:**

Bruker APEXII CCD diffractometer	2547 reflections with *I* > 2σ(*I*)
φ and ω scans	*R*_int_ = 0.038
Absorption correction: multi-scan (SADABS; Bruker, 2003)	θ_max_ = 26.4°, θ_min_ = 2.9°
*T*_min_ = 0.911, *T*_max_ = 0.946	*h* = −17→16
11687 measured reflections	*k* = −16→15
3156 independent reflections	*l* = −10→10

### (E)-1-(4-Chlorophenyl)-2-[2,2-dichloro-1-(4-nitrophenyl)ethenyl]diazene (II) Refinement

**Table:**

Refinement on *F*^2^	Hydrogen site location: inferred from neighbouring sites
Least-squares matrix: full	H-atom parameters constrained
*R*[*F*^2^ > 2σ(*F*^2^)] = 0.037	*w* = 1/[σ^2^(*F*_o_^2^) + (0.0419*P*)^2^ + 0.3296*P*] where *P* = (*F*_o_^2^ + 2*F*_c_^2^)/3
*wR*(*F*^2^) = 0.091	(Δ/σ)_max_ < 0.001
*S* = 1.04	Δρ_max_ = 0.18 e Å^−^^3^
3156 reflections	Δρ_min_ = −0.25 e Å^−^^3^
199 parameters	Absolute structure: Flack *x* determined using 1011 quotients [(*I*^+^)-(*I*^-^)]/[(*I*^+^)+(*I*^-^)] (Parsons *et al.*, 2013).
1 restraint	Absolute structure parameter: 0.14 (3)

### (E)-1-(4-Chlorophenyl)-2-[2,2-dichloro-1-(4-nitrophenyl)ethenyl]diazene (II) Special details

**Table:**

Geometry. All esds (except the esd in the dihedral angle between two l.s. planes) are estimated using the full covariance matrix. The cell esds are taken into account individually in the estimation of esds in distances, angles and torsion angles; correlations between esds in cell parameters are only used when they are defined by crystal symmetry. An approximate (isotropic) treatment of cell esds is used for estimating esds involving l.s. planes.

### (E)-1-(4-Chlorophenyl)-2-[2,2-dichloro-1-(4-nitrophenyl)ethenyl]diazene (II) Fractional atomic coordinates and isotropic or equivalent isotropic displacement parameters (Å^2^)

**Table:**

	*x*	*y*	*z*	*U*_iso_*/*U*_eq_	
C1	0.9044 (2)	0.0035 (2)	0.3115 (4)	0.0400 (8)	
C2	0.8753 (3)	−0.0900 (2)	0.3650 (5)	0.0507 (9)	
H2	0.813001	−0.099076	0.403349	0.061*	
C3	0.9385 (3)	−0.1694 (2)	0.3615 (6)	0.0569 (10)	
H3	0.919592	−0.231831	0.399107	0.068*	
C4	1.0294 (3)	−0.1555 (3)	0.3023 (5)	0.0532 (10)	
C5	1.0584 (3)	−0.0645 (3)	0.2434 (6)	0.0646 (12)	
H5	1.119552	−0.056954	0.199546	0.077*	
C6	0.9962 (3)	0.0155 (3)	0.2498 (6)	0.0607 (11)	
H6	1.015836	0.077758	0.212526	0.073*	
C7	0.6941 (2)	0.1515 (2)	0.3415 (4)	0.0376 (7)	
C8	0.6001 (2)	0.1294 (2)	0.3491 (6)	0.0480 (8)	
C9	0.7299 (2)	0.2569 (2)	0.3447 (5)	0.0364 (6)	
C10	0.7644 (3)	0.3003 (3)	0.2053 (4)	0.0464 (9)	
H10	0.766518	0.263150	0.111319	0.056*	
C11	0.7955 (3)	0.3983 (3)	0.2056 (4)	0.0461 (9)	
H11	0.818819	0.427923	0.112661	0.055*	
C12	0.7913 (2)	0.4511 (2)	0.3468 (5)	0.0386 (7)	
C13	0.7591 (3)	0.4093 (3)	0.4853 (5)	0.0530 (10)	
H13	0.757981	0.446192	0.579602	0.064*	
C14	0.7280 (3)	0.3110 (3)	0.4834 (5)	0.0507 (10)	
H14	0.705639	0.281565	0.577118	0.061*	
N1	0.8441 (2)	0.09023 (19)	0.3159 (4)	0.0446 (7)	
N2	0.75630 (18)	0.06882 (17)	0.3339 (4)	0.0408 (6)	
N3	0.8211 (2)	0.55693 (19)	0.3468 (5)	0.0487 (7)	
O1	0.8400 (3)	0.5963 (2)	0.2197 (4)	0.0754 (10)	
O2	0.8259 (3)	0.6004 (2)	0.4729 (4)	0.0879 (13)	
Cl1	1.10869 (9)	−0.25630 (8)	0.2991 (2)	0.0857 (4)	
Cl2	0.55690 (6)	0.00980 (6)	0.3445 (2)	0.0727 (3)	
Cl3	0.51218 (7)	0.21874 (7)	0.3590 (2)	0.0751 (4)	

### (E)-1-(4-Chlorophenyl)-2-[2,2-dichloro-1-(4-nitrophenyl)ethenyl]diazene (II) Atomic displacement parameters (Å^2^)

**Table:**

	*U*^11^	*U*^22^	*U*^33^	*U*^12^	*U*^13^	*U*^23^
C1	0.0437 (17)	0.0277 (14)	0.048 (2)	−0.0013 (12)	−0.0011 (15)	−0.0030 (14)
C2	0.0481 (18)	0.0381 (16)	0.066 (2)	−0.0012 (14)	0.008 (2)	0.008 (2)
C3	0.062 (2)	0.0325 (16)	0.076 (3)	−0.0010 (15)	0.003 (2)	0.007 (2)
C4	0.053 (2)	0.0379 (18)	0.069 (3)	0.0094 (15)	−0.0048 (18)	−0.0087 (18)
C5	0.046 (2)	0.054 (2)	0.093 (3)	0.0019 (18)	0.012 (2)	0.001 (2)
C6	0.052 (2)	0.039 (2)	0.091 (3)	−0.0075 (17)	0.008 (2)	0.004 (2)
C7	0.0461 (16)	0.0271 (13)	0.0397 (17)	0.0000 (12)	−0.0005 (18)	−0.0028 (17)
C8	0.0471 (17)	0.0284 (14)	0.069 (2)	−0.0008 (13)	−0.004 (2)	0.0006 (19)
C9	0.0361 (14)	0.0294 (13)	0.0437 (17)	0.0023 (11)	−0.0006 (16)	−0.0014 (19)
C10	0.065 (2)	0.037 (2)	0.037 (2)	−0.0058 (17)	0.0002 (18)	−0.0045 (16)
C11	0.061 (2)	0.038 (2)	0.039 (2)	−0.0090 (17)	0.0010 (17)	0.0031 (16)
C12	0.0427 (16)	0.0273 (13)	0.0458 (18)	−0.0003 (12)	−0.0036 (19)	−0.0010 (19)
C13	0.075 (3)	0.039 (2)	0.046 (2)	−0.0076 (19)	0.0109 (19)	−0.0099 (17)
C14	0.073 (3)	0.037 (2)	0.042 (2)	−0.0102 (18)	0.0089 (19)	−0.0038 (17)
N1	0.0476 (15)	0.0295 (12)	0.057 (2)	−0.0009 (11)	0.0003 (14)	−0.0009 (14)
N2	0.0427 (14)	0.0313 (12)	0.0485 (16)	−0.0006 (10)	−0.0025 (15)	−0.0011 (15)
N3	0.0533 (16)	0.0323 (13)	0.061 (2)	−0.0051 (12)	0.001 (2)	0.002 (2)
O1	0.118 (3)	0.0418 (17)	0.067 (2)	−0.0227 (18)	0.0012 (19)	0.0106 (17)
O2	0.149 (4)	0.0453 (19)	0.069 (2)	−0.028 (2)	0.013 (2)	−0.0211 (18)
Cl1	0.0767 (7)	0.0574 (6)	0.1230 (12)	0.0296 (5)	−0.0039 (7)	−0.0084 (7)
Cl2	0.0513 (5)	0.0375 (4)	0.1292 (10)	−0.0109 (3)	−0.0096 (7)	0.0036 (7)
Cl3	0.0456 (5)	0.0450 (5)	0.1348 (11)	0.0067 (4)	−0.0006 (7)	0.0016 (7)

### (E)-1-(4-Chlorophenyl)-2-[2,2-dichloro-1-(4-nitrophenyl)ethenyl]diazene (II) Geometric parameters (Å, º)

**Table:**

C1—C6	1.383 (5)	C8—Cl3	1.708 (3)
C1—C2	1.387 (5)	C9—C14	1.368 (5)
C1—N1	1.430 (4)	C9—C10	1.387 (5)
C2—C3	1.377 (5)	C10—C11	1.379 (5)
C2—H2	0.9300	C10—H10	0.9300
C3—C4	1.366 (6)	C11—C12	1.377 (5)
C3—H3	0.9300	C11—H11	0.9300
C4—C5	1.373 (6)	C12—C13	1.362 (6)
C4—Cl1	1.740 (4)	C12—N3	1.473 (4)
C5—C6	1.374 (6)	C13—C14	1.382 (5)
C5—H5	0.9300	C13—H13	0.9300
C6—H6	0.9300	C14—H14	0.9300
C7—C8	1.339 (4)	N1—N2	1.259 (4)
C7—N2	1.404 (4)	N3—O2	1.207 (5)
C7—C9	1.493 (4)	N3—O1	1.214 (4)
C8—Cl2	1.708 (3)		
			
C6—C1—C2	119.5 (3)	C14—C9—C10	119.9 (3)
C6—C1—N1	117.0 (3)	C14—C9—C7	120.5 (3)
C2—C1—N1	123.5 (3)	C10—C9—C7	119.6 (3)
C3—C2—C1	120.2 (3)	C11—C10—C9	120.3 (3)
C3—C2—H2	119.9	C11—C10—H10	119.9
C1—C2—H2	119.9	C9—C10—H10	119.9
C4—C3—C2	119.4 (3)	C12—C11—C10	118.4 (3)
C4—C3—H3	120.3	C12—C11—H11	120.8
C2—C3—H3	120.3	C10—C11—H11	120.8
C3—C4—C5	121.3 (3)	C13—C12—C11	122.2 (3)
C3—C4—Cl1	118.9 (3)	C13—C12—N3	119.1 (3)
C5—C4—Cl1	119.7 (3)	C11—C12—N3	118.7 (3)
C4—C5—C6	119.4 (4)	C12—C13—C14	118.9 (3)
C4—C5—H5	120.3	C12—C13—H13	120.6
C6—C5—H5	120.3	C14—C13—H13	120.6
C5—C6—C1	120.2 (4)	C9—C14—C13	120.4 (4)
C5—C6—H6	119.9	C9—C14—H14	119.8
C1—C6—H6	119.9	C13—C14—H14	119.8
C8—C7—N2	115.3 (3)	N2—N1—C1	112.6 (2)
C8—C7—C9	122.1 (3)	N1—N2—C7	114.9 (2)
N2—C7—C9	122.7 (3)	O2—N3—O1	123.0 (3)
C7—C8—Cl2	123.2 (2)	O2—N3—C12	118.5 (3)
C7—C8—Cl3	122.9 (2)	O1—N3—C12	118.4 (3)
Cl2—C8—Cl3	113.90 (19)		
			
C6—C1—C2—C3	2.1 (7)	C7—C9—C10—C11	178.5 (3)
N1—C1—C2—C3	−178.0 (4)	C9—C10—C11—C12	0.0 (6)
C1—C2—C3—C4	−1.1 (7)	C10—C11—C12—C13	1.1 (6)
C2—C3—C4—C5	−1.3 (7)	C10—C11—C12—N3	−177.8 (3)
C2—C3—C4—Cl1	179.8 (3)	C11—C12—C13—C14	−1.1 (6)
C3—C4—C5—C6	2.6 (8)	N3—C12—C13—C14	177.7 (3)
Cl1—C4—C5—C6	−178.5 (4)	C10—C9—C14—C13	0.9 (6)
C4—C5—C6—C1	−1.6 (8)	C7—C9—C14—C13	−178.5 (4)
C2—C1—C6—C5	−0.8 (7)	C12—C13—C14—C9	0.1 (6)
N1—C1—C6—C5	179.4 (4)	C6—C1—N1—N2	162.9 (4)
N2—C7—C8—Cl2	−1.8 (6)	C2—C1—N1—N2	−17.0 (5)
C9—C7—C8—Cl2	179.6 (3)	C1—N1—N2—C7	179.0 (3)
N2—C7—C8—Cl3	−179.9 (3)	C8—C7—N2—N1	175.4 (4)
C9—C7—C8—Cl3	1.4 (6)	C9—C7—N2—N1	−5.9 (5)
C8—C7—C9—C14	73.1 (5)	C13—C12—N3—O2	7.8 (5)
N2—C7—C9—C14	−105.4 (4)	C11—C12—N3—O2	−173.3 (4)
C8—C7—C9—C10	−106.3 (5)	C13—C12—N3—O1	−172.4 (4)
N2—C7—C9—C10	75.2 (5)	C11—C12—N3—O1	6.5 (5)
C14—C9—C10—C11	−0.9 (6)		

### (E)-1-(4-Chlorophenyl)-2-[2,2-dichloro-1-(4-nitrophenyl)ethenyl]diazene (II) Hydrogen-bond geometry (Å, º)

*Cg*2 is the centroid of the C9–C14 ring.

**Table:**

*D*—H···*A*	*D*—H	H···*A*	*D*···*A*	*D*—H···*A*
C8—Cl3···*Cg*2^i^	1.71 (1)	3.62 (1)	4.703 (3)	120 (1)

Symmetry code: (i) *x*−1/2, −*y*+1/2, *z*.
